# Terpene-augmented novasomal carriers for trans-tympanic drug delivery: a comprehensive optimization and in vivo evaluation

**DOI:** 10.1007/s00210-025-04659-x

**Published:** 2025-11-17

**Authors:** Sadek Ahmed, Ali Fayez, Heba Attia, Doaa Ahmed El-Setouhy

**Affiliations:** 1 Department of Pharmaceutics and Industrial Pharmacy, Faculty of Pharmacy, Cairo University, Kasr El-Aini, Cairo, Egypt P.O. Box 11562,; 2 Ministry of Health, Jeddah, Kingdom of Saudi Arabia, Jeddah, Saudi Arabia; 3 Department of Microbiology and Immunology, Faculty of Pharmacy, Cairo University, Cairo, Egypt

**Keywords:** Levofloxacin, Novasomes, Rheology, In vivo permeation, Biofilm inhibition

## Abstract

Otitis media (OM) is a frequent infectious condition that affects the middle ear especially in children. The purpose of this project was to create fenchone-augmented novasomes to enhance the trans-tympanic penetration of levofloxacin (LFX) and improve its antibacterial efficacy. Novasomes were formulated using the ethanol injection technique and optimized using a 2^3^ factorial design. The factors analyzed included the stearic acid to drug ratio (A), cholesterol to fenchone ratio (B), and surfactant concentration (%) (C). The optimization feature of Design-Expert® software was utilized to identify the optimal formulation by minimizing particle size (PS) and poly-dispersity index (PDI) while maximizing entrapment efficiency (EE%) and the absolute value of the zeta potential (ZP). The best formulation achieved a desirability of 0.964, with an EE% of 73.39%, a PS of 179.25 nm, and a ZP of − 31.95 mV. The formulation will be subjected to additional evaluations, including in vitro, ex vivo, microbiological, and in vivo testing. A thorough in vitro analysis revealed a biphasic release profile, significant stability, and a spherical morphology. Novasomes exhibited greater ex vivo permeation and superior flux values compared to the LFX solution. The optimum formula demonstrated high otic tolerance. The optimized formula demonstrated markedly enhanced antibacterial activity, with significantly lower minimum inhibitory concentration (MIC) and minimum bactericidal concentration (MBC) values against *Staphylococcus aureus* and *Pseudomonas aeruginosa* compared to the drug solution. Moreover, it exhibited superior biofilm inhibition, even at sub-MIC concentrations, underscoring its efficacy. These findings highlight the potential of LFX-loaded novasomes as an efficient non-invasive method for managing middle ear infections.

## Introduction

The auditory system is a delicate and highly organized structure responsible for hearing and maintaining balance. It consists of three anatomically distinct regions: the outer ear, middle ear, and inner ear. The outer ear comprises the pinna and the external auditory canal, extending to the tympanic membrane (TM), a thin lipid- and keratin-rich barrier that plays a crucial role in sound conduction. The Eustachian tube, which links the middle ear to the pharynx, helps equilibrate air pressure across the TM. The inner ear includes the cochlea and vestibular system, both essential for sensory functions related to hearing and equilibrium (Hao & Li 2019). Acute otitis media (AOM), an infection of the middle ear ranks as the second most common diagnosis in pediatric emergency settings after upper respiratory tract infections. AOM predominantly affects children between 6 and 24 months of age and is primarily caused by pathogens such as *Streptococcus pneumoniae*, *Haemophilus influenzae*, *Staphylococcus aureus*, and *Streptococcus pyogenes* (Al-Mahallawi et al. 2014; Jaudoin et al. 2021; Z. Zhang et al. 2021). The infection results from microbial migration via the Eustachian tube, leading to fluid buildup and inflammation. AOM affects approximately 80% of children by the age of five. Epidemiological studies further indicate that many children, particularly in the USA, may experience up to six episodes by the age of seven (Ahmed et al. 2025f). This recurrent and widespread nature of AOM underscores its clinical significance and highlights the urgent need for more effective, localized, and targeted therapeutic strategies to reduce disease burden and minimize complications associated with repeated antibiotic use (El Feghaly et al. 2023).

Conventional treatment typically involves systemic administration of broad-spectrum antibiotics, which may lead to undesirable side effects and contribute to antimicrobial resistance. Consequently, local otic delivery has emerged as a promising alternative. However, several physiological barriers—such as the tympanic membrane, the blood–inner ear barrier, and limited drug permeability through the round and oval windows severely restrict drug bioavailability at the target site (Magdy et al. 2022; Yang et al. 2017; Zou et al. 2016).

Numerous nanocarrier systems, including glycerosomes (Magdy et al. 2022), transferosomes (Al-Mahallawi et al. 2014), and glycerylated vesicles (Adwan et al. 2015), have been explored to overcome these challenges and enhance the otic delivery of antibiotics. Novasomes are an emerging class of vesicular drug delivery systems originally developed by IGI Laboratories (NOVAVAX) to enhance the performance and stability of conventional carriers. Structurally, they represent an advanced form of liposomes or niosomes, typically composed of cholesterol, free fatty acids (FFAs), and polyoxyethylene fatty acid monoesters, providing improved encapsulation capacity and controlled drug release characteristics (Farag et al. 2025; Mosallam et al. 2021a). Stearic acid was selected as the lipid component in the formulation due to its well-established role in enhancing the stability and structural integrity of lipid-based vesicles. As a long-chain saturated fatty acid, stearic acid contributes to the rigidity of the bilayer membrane, thereby improving the mechanical strength and physical stability of the vesicular system. Additionally, it is biocompatible, non-toxic, and widely used in topical and mucosal formulations (Singh et al. 2020). Its incorporation has been shown to enhance drug encapsulation efficiency and provide a more sustained release profile, both of which are advantageous for localized drug delivery through the trans-tympanic route (Ahmed et al. 2022a).

Fenchone, a naturally occurring monoterpene, has shown promising potential as a permeation enhancer in drug delivery systems targeting mucosal barriers. Its lipophilic nature allows it to interact intimately with biological membranes, where it disrupts the organized structure of lipid bilayers. This disruption increases membrane fluidity and reduces the tight packing of lipid molecules, thereby facilitating the translocation of encapsulated drugs. Additionally, fenchone can form hydrogen bonds with membrane components, further destabilizing the bilayer and enhancing permeability. These synergistic effects contribute to improved trans-tympanic drug transport, ultimately boosting local drug bioavailability and therapeutic performance (Ahmed et al. 2025e; Albash et al. 2021).

Levofloxacin (LFX), a third-generation fluoroquinolone, exhibits potent broad-spectrum antibacterial activity and has shown clinical efficacy against pathogens commonly implicated in AOM, including *S. aureus* and *P. aeruginosa*. Prior studies have reported its favorable stability and strong antimicrobial performance in otic infections (A. A. Abdelbary et al. 2019; He & Li 2022). Levofloxacin (LFX) is commonly administered in the treatment of bacterial infections affecting the eye, often prescribed in the form of eye drops at frequent intervals initially every 1–2 h for the first few days, followed by reduced dosing every 4–5 h thereafter (A. A. Abdelbary et al. 2019).

Despite these advances, the potential of combining LFX with fenchone-enriched novasomes to facilitate non-invasive, trans-tympanic delivery has not been explored to date. Fenchone, as known membrane permeation enhancers, could further improve drug flux across the TM, making them ideal candidates for otic drug delivery systems (Ahmed et al. 2025e; Nemr et al. 2024). This forms the central hypothesis of the current study: that incorporating fenchone into a nanoscale novasomal system will significantly enhance trans-tympanic permeability and antibacterial efficacy of LFX, while maintaining safety for otic tissues. Accordingly, the present work aims to develop and optimize a fenchone-augmented novasomal formulation using a 2^3^ full factorial design to investigate the effects of stearic acid:drug ratio, cholesterol:fenchone ratio, and surfactant concentration on key formulation attributes. The optimized formulation was evaluated through a comprehensive set of in vitro, ex vivo, microbiological, and in vivo studies to assess its drug release behavior, antimicrobial activity, tympanic membrane permeation, and histological safety. The study further seeks to establish a rationale for using fenchone-loaded novasomes as a clinically relevant, non-invasive strategy for managing otitis media.

## Materials and methods

### Materials

LFX, provided by SEDICO Pharmaceutical Co. (Cairo, Egypt); fenchone, Span 65, cholesterol, Rhodamine B, and dialysis membrane (14,000 Da) were obtained from Sigma Aldrich Chemical Co. (St. Louis, MO, USA); and ethanol (95%) and formaldehyde from El-Nasr Pharmaceutical Chemicals Co. (Cairo, Egypt). All other chemicals and solvents used were of analytical grade and employed without additional purification.

### Experimental animals

Mature male white rabbits, each weighing approximately 2 ± 0.5 kg, were used in this study. The animals were housed individually under controlled conditions (25 ± 2 °C temperature, 12-h light/dark cycle) with access to tap water and a standard commercial diet. Ethical approval for animal use was obtained from the Research Ethics Committee for Experimental and Clinical Studies at the Faculty of Pharmacy, Cairo University, Egypt (Approval no. PI 3733). Animal care and handling followed the US National Institutes of Health’s Guide for the Care and Use of Laboratory Animals (NIH Publication No. 85–23, revised 2011) and complied with the ARRIVE guidelines for reporting in vivo experiments.

### Methods

#### Experimental design

LFX-loaded novasomes were developed using a 2^3^ factorial design. There were two levels for every independent variable under investigation, yielding eight runs (T1–T8). These variables were the drug:stearic acid ratio (A), cholesterol:fenchone ratio (B), and surfactant concentration (C). The preliminary findings guided the choice of levels under investigation. The optimal formula was determined by analyzing the responses (dependent variables): EE% (Y1), PS (Y2), PDI (Y3), and ZP (Y4). The exact values of the levels under study and the dependent variables’ desirability are shown in Table 1. Design-Expert® software (Stat-Ease, Inc., Minneapolis, Minnesota, USA) was utilized to determine each response’s significance (Ahmed et al. 2025e; El Hassab et al. 2025).

**Table 1:** Factorial levels of studied independent variables together with measured responses and their desirability constraints

Factor (independent variable)	Level	
	** − 1**	** + 1**
A: Stearic acid:drug ratio B: Cholesterol:fenchone ratio C: Surfactant concentration (%)	5:1 1:1 0.25	10:1 2:1 0.5
**Response (dependent variable)**	**Desirability constraints**	
Y1: EE% Y2: PS (nm) Y3: PDI Y4: ZP (absolute value) (mV)	Maximize Minimize Minimize Maximize	

*EE%*, percent entrapment efficiency; *PDI*, poly-dispersity index; *PS*, particle size; *ZP*, zeta potential

#### Preparation of LFX-loaded novasomes

A modified ethanol injection method was used to fabricate LFX-loaded novasomes (Su et al. 2024). In summary, LFX (10 mg), fenchone (50 mg), and specific amounts of cholesterol, surfactants, and stearic acid were precisely weighed and mixed in ethanol (10 mL), heated at 60 °C in a water bath (Crest Ultrasonics Corp., Trenton, USA). The resulting ethanol mixture was then added slowly to twice its volume of distilled water then continuously stirred at the same temperature until complete evaporation of ethanol. The first appearance of turbidity indicates that the novasomes had been formulated (Abdurrahman M. Fahmy et al. 2025). The resulting novasomal dispersions were sonicated (Ultra Sonicator, model LC 60/H, Elma, Singen, Germany) at 25 ± 2 °C for 20 min to reduce particle size and were then stored at 4 °C for future use (Albash et al. 2022b; Mosallam et al. 2021b). Table 2 outlines the details of the LFX-encapsulated novasomal formulations (T1–T8), arranged randomly. The absence of any detectable ethanol odor indicated that the residual solvent had been entirely eliminated (Ahmed et al. 2025d).

**Table 2:** Composition of LFX-loaded novasomes with their measured responses (*n* = 3 ± SD)

**Formula**	**Factors**						
	**A: stearic acid:drug**	**B: cholesterol:fenchone**	**C: surfactant concentration (%)**	**Y1: EE%** **(mean ± SD)**	**Y2: PS (nm)** **(mean ± SD)**	**Y3: PDI** **(mean ± SD)**	**Y4: ZP (mV)** **(mean ± SD)**
**F1**	5:1	1:1	0.5	64.71 ± 3.12	158.35 ± 6.86	0.26 ± 0.04	− 25.50 ± 1.84
**F2**	10:1	2:1	0.25	61.31 ± 0.69	152.00 ± 2.40	0.17 ± 0.01	− 24.25 ± 1.63
**F3**	5:1	2:1	0.25	57.60 ± 0.21	145.95 ± 2.05	0.42 ± 0.07	− 22.90 ± 1.13
**F4**	5:1	1:1	0.25	53.87 ± 0.72	133.90 ± 1.41	0.16 ± 0.02	− 20.80 ± 0.28
**F5**	10:1	2:1	0.5	73.39 ± 7.49	179.25 ± 3.61	0.24 ± 0.02	− 31.95 ± 0.21
**F6**	10:1	1:1	0.25	56.62 ± 0.26	142.75 ± 0.92	0.12 ± 0.02	− 22.85 ± 1.91
**F7**	10:1	1:1	0.5	66.85 ± 5.15	161.15 ± 3.46	0.26 ± 0.01	− 28.25 ± 0.92
**F8**	5:1	2:1	0.5	69.74 ± 6.94	172.60 ± 3.96	0.31 ± 0.01	− 30.60 ± 1.41

*LFX*, levofloxacin; *EE%,* percent entrapment efficiency; *PDI*, poly-dispersity index; *PS*, particle size; *ZP*, zeta potential

#### In vitro characterization of LFX-loaded novasomes Entrapment efficiency (EE%)

##### Entrapment efficiency (EE%)

The entrapment efficiency (EE%) of LFX was assessed using a non-direct spectrophotometric analysis (Shimadzu, model UV-1601 PC, Kyoto, Japan) to measure the free LFX in the supernatant (El Hassab et al. 2025; Younes et al. 2024). Briefly, the optimized preparation was subjected to centrifugation at 21,000 rpm for 1 h at 4 °C. The concentration of free LFX was then determined at a pre-calibrated wavelength of 288 nm (*n* = 3; *R*^2^ = 0.9998). The EE% was determined using the equation below (Ahmed et al. 2025c, d):

EE% = × 100 (Eq. 1).

1 $$\frac{(\text{Quantity of LFX used }-\text{ Quantity of unentrapped LFX})}{\text{Quantity of LFX used}}$$

##### Measurement of particle size (PS), polydispersity index (PDI), and zeta potential (ZP)

A translucent dispersion was obtained by diluting each formulation with an appropriate volume of distilled water. The particle size (PS), zeta potential (ZP), and polydispersity index (PDI) were then measured using a Zetasizer (Model ZEN3600, Malvern Instruments Ltd., Worcestershire, UK). Every measurement was made in three duplicates at 25 °C (Albash et al. 2025a, c; Ibrahim et al. 2024a).

#### Formulation optimization and validation

The numerical optimization module of Design-Expert software (Stat-Ease Inc., Minneapolis, MN, USA) was employed to determine the optimal formulation. Briefly, the collected experimental responses were statistically analyzed using analysis of variance (ANOVA). The formulation with the highest desirability index was selected by maximizing encapsulation efficiency (EE%) and the absolute value of zeta potential (ZP), while minimizing the polydispersity index (PDI) and particle size (PS). The percentage deviation was computed, and the observed and predicated results were compared for validation (Ahmed et al. 2025a; Albash et al. 2021; Tawfik et al. 2025). A low percentage deviation reflects the robustness and reliability of the optimization process, indicating a high level of accuracy and consistency in the applied optimization strategy.

#### In vitro characterization of the optimized LFX-loaded formula

##### Fourier transform infrared spectroscopy (FTIR)

FTIR spectra were recorded for the lyophilized optimal formula, cholesterol, stearic acid, pure LFX, and Span 65 using a Fourier transform infrared spectrophotometer (Model 22, Bruker, Coventry, UK). The samples were prepared as potassium bromide (KBr) pellets, and the analysis was conducted at 25 ± 2 °C within the 4000–500 cm⁻^1^ range (Ahmed et al. 2024b, a; Elgendy et al. 2023).

##### Transmission electron microscopy (TEM)

The optimal formulation was diluted with double-distilled water, applied to copper grids covered with carbon, and stained with a 2% solution of phosphotungstic acid. The morphological characteristics were then analyzed using a TEM (JEOL, Tokyo, Japan) (Albash et al. 2025b; Carol Yousry et al. 2023).

##### In vitro drug release profiling and kinetic modeling

Although the physiological pH of the tympanic membrane ranges from 4.2 to 5.6, a pH of 7.4 was selected to simulate the alkaline conditions associated with acute otitis media, where local inflammation and exudate accumulation have been reported to elevate the pH. This approach has been similarly adopted in previous studies to better mimic the pathological environment (Al-Mahallawi et al. 2014, 2017; Younes et al. 2024). The dialysis method was used to assess the in vitro release profile of the optimal formulation. Dialysis membrane was soaked overnight in phosphate-buffered saline with pH 7.4, and a dialysis bag containing 1 mg of LFX from either the optimal formulation or LFX solution was placed in 50 mL of release medium at 37 ± 0.5 °C and 100 rpm (Ahmed et al. 2025a, c). Samples were withdrawn at 0.5, 1, 2, 4, 6, and 8 h and replaced with a freshly prepared release medium to maintain sink conditions (Ahmed et al. 2024a). The percentage of LFX released was measured spectrophotometrically, and the data were analyzed using kinetic models, including zero-order, first-order, and second-order kinetics, as well as Higuchi’s diffusion models, to interpret the release behavior (Abdelhakeem et al. 2025; Ahmed et al. 2025b).

##### Rheological characterization and flow behavior analysis

The optimal formulation was evaluated rheologically using a Brookfield viscometer (spindle CPE-41) at 25 ± 1 °C. On the plate, 0.5-g sample was placed with variable rotational speeds between 0.5 and 100 rpm, with a 10-s pause between speed changes (A. A. Nemr et al. 2021). Valid results required the torque to be within the 10 to 100% range. A graph was plotted to show the relationship between viscosity, shear rate, and shear stress, and the power law model was applied to analyze the rheological behavior of the optimal formula using the following formula (Younes et al. 2025):.

2 $$T=K\gamma^{n\cdot}$$

The consistency index (*K*) and flow index (*n*) are key parameters for characterizing the rheological behavior of fluids. In shear-thinning systems, *n* ranges between 0 and 1; in dilatant systems, it exceeds 1; and in Newtonian systems, it equals 1. Non-Newtonian behavior can be modeled using equations such as those of Bingham, Casson, and Carreau, with the most suitable model determined by comparing their respective *R*^2^ values (A. M. Fahmy et al. 2018).

##### Stability study

The optimal formulation was stored at a temperature of 4–8 °C for 3 months, and stability testing was performed to assess its properties and activities (Sayed et al. 2021). Physical appearance, particle size (PS), entrapment efficiency (EE%), zeta potential (ZP), and release profile were re-evaluated and compared with the fresh formula. EE%, PS, and ZP were compared using one-way ANOVA, while similarity factor “ƒ2” was used to compare the release profiles using the following formula (Adel et al. 2021):

3 $${f}_{2}=50.\mathrm{log}\{[1+\left(\frac{1}{n}\right){\sum }_{t=1}^{n}{\left({R}_{t}-{T}_{t}\right)}^{2}{]}^{-0.5}.100$$

At time *t*, *R*_*t*_ and *T*_*t*_ represent the amount of LFX released before and after storage, respectively. To assure similarity, the value of the similarity factor “ƒ2” should be between 50 and 100 (G. A. Abdelbary et al. 2016; Hegazy et al. 2025).

#### Ex vivo evaluation of trans-tympanic drug permeation

Male albino rabbits (2 ± 0.5 kg) were anesthetized with ketamine and xylazine before euthanasia (Ahmed et al. 2024a, 2025f). The tympanic membranes (TMs) were excised as mentioned by Khoo et al. (Khoo et al. 2013). After removing the rabbit bulla, the medial surface of the TM was placed in contact with the receptor medium and suspended vertically using thread in a beaker containing 25 mL of phosphate-buffered saline (pH 7.4). To maintain a temperature of 37 °C and agitation, a magnetic stirrer was set to 100 rpm. The lateral surface of the TM was then coated with 1 mg of LFX from either the optimal formula or LFX solution (A. A. Abdelbary et al. 2019; Al-Mahallawi et al. 2017). Samples were collected at 1, 2, 4, 6, 8, and 10 h, with the receptor medium being replaced to maintain sink conditions (Ahmed et al. 2022b; Al-Mahallawi et al. 2014). LFX concentration in the samples was measured spectrophotometrically. Parameters, including the cumulative amount of LFX permeated after 10 h (Q₁₀h), the enhancement ratio (ER), and the maximum flux (Jmax), were calculated. A penetration profile was constructed by plotting LFX concentration (µg/cm^2^) against time (h). The corresponding equations for ER and Jmax were applied (ElMeshad & Mohsen 2016; Elsadek et al. 2024):

4 $$J_{max}\cdot=\cdot\frac{Amount\;of\;drug\;permented}{Time\times Area}$$

5 $$ER\cdot=\cdot\frac{Jmax\;of\;formulation}{Jmax\;of\;drug\;solution}$$

#### Microbiological analysis

##### Determination of the antibacterial activity of both the drug solution and its optimum formula

To evaluate the antibacterial effectiveness of the optimal formulation versus the drug solution, minimum inhibitory concentration (MIC) and minimum bactericidal concentration (MBC) tests were performed. The MIC was determined using the broth microdilution method in accordance with the Clinical and Laboratory Standards Institute guidelines (Humphries et al. 2018). Twofold serial dilutions of the drug and optimal formula (concentrations range between 500–0.244 µg/mL) were prepared in 100 µL double-strengthMuller-Hinton Broth (MHB) and dispensed into a 96-well plate with bacterial suspensions (20 µL) of *Staphylococcus aureus* (USA300) or *Pseudomonas aeruginosa* PAO1 (10^5^–10⁶ CFU/mL). Controls included positive, blank, and negative controls to confirm bacterial growth, investigate any possible antibacterial activity of the recipient, and to check the sterility. Plates were incubated for 24 h at 35 °C ± 2 °C, then measured at 600 nm, and the MIC was identified as the lowest concentration showing no observable growth. Biological and technical triplicates (*n* = 9) were performed.

To determine the minimum bactericidal concentration (MBC), twofold serial dilutions of the drug and formula were combined with 20 µL of bacterial suspension (10^5^–10⁶ CFU/mL) and incubated in 96-well plates at 35 °C ± 2 °C for 24 h. Following incubation, 10 µL of the bacterial inoculum and the diluted solutions mixture were transferred to the surface of Muller-Hinton Agar (MHA) plates and incubated at 35 °C ± 2 °C for an additional 24 h. The MBC was identified as the lowest concentration that showed no visible bacterial growth on the MHA plates. This experiment was performed in both biological and technical triplicates (*n* = 9) (Humphries et al. 2018).

##### Determination of the biofilm inhibition activity

To assess the potential enhancement in biofilm inhibition activity of the optimal formulation relative to the drug solution, 50 μL of a 10⁸ CFU/mL bacterial inoculum was added to 50 μL of either the drug solution or the optimal formulation in sterile, non-pyrogenic polystyrene, flat-bottom 96-well culture plates. The final concentrations corresponded to ^1^/_16_, ^1^/_8_, ^1^/_4_ and ^1^/_2_ X, where X, represents the determined MIC. The experiment includes positive, negative, and blank controls (Haney et al. 2021).

After incubating the microtiter plates at 35 ± 2 °C for 24 h, the optical density (OD600) was measured using an automated spectrophotometric plate reader (Biotek, Synergy 2, USA). The supernatants were discarded, and the wells were washed twice with sterile saline (pH 7.4) before drying. The dried biofilms were stained with 125 μL of 0.5% w/v crystal violet for 30 min at room temperature. After staining, the plates were washed three times with sterile distilled water and dried thoroughly. To solubilize the stained biofilms, 150 μL of 95% ethanol was added to each well, and the plates were incubated for 15 min with shaking. The optical density (OD570) of the solubilized biofilm was then measured, normalized to the OD600 of the planktonic bacterial inoculum, and the biofilm inhibition percentage was calculated. The experiment was repeated three times with technical replicates. The percentage of biofilm inhibition is calculated using the following formula:

6 $$\frac{OD\;Control-OD\;Test}{OD\;Control}\times100$$

#### In vivo evaluation of the optimized formulation

##### Assessment of the histopathological changes

To assess the otic safety of the optimized formulation, a histopathological evaluation was conducted using healthy male albino rabbits (2 ± 0.5 kg). Sterile normal saline served as the negative control. The optimized formulation (100 µL) was instilled into the right ear three times daily for seven consecutive days, whereas the left ear received an equivalent volume of normal saline for comparative assessment (A. A. Abdelbary et al. 2019). At the end of the treatment period, animals were anesthetized and humanely euthanized in accordance with ethical guidelines. The tympanic membranes (TMs) were carefully excised and immediately fixed in 10% v/v formalin for histological analysis. Tissue specimens were rinsed with double-distilled water, dehydrated through ascending concentrations of ethanol, and cleared in xylene. The samples were embedded in paraffin wax at 56 °C for 24 h. Tissue sections were then prepared using a rotary microtome, mounted on clean glass slides, deparaffinized, and stained with hematoxylin and eosin. Subsequently, the slides were examined under a light microscope to evaluate morphological alterations and detect any signs of tissue damage. (R. Albash et al. 2024).

##### In vivo permeation

To assess the permeation capacity of the optimal formulation, Rhodamine B (RhB) was employed as a surrogate for LFX due to its suitability for evaluating deep tissue penetration, which is essential for effective antibacterial activity. The optimal formulation was prepared with a 0.1% w/w RhB concentration to determine the penetration depth of both the RhB-loaded optimal formulation and the RhB-loaded aqueous solution (negative control). Confocal laser scanning microscopy (CLSM) was utilized for the analysis (LSM 710; Carl Zeiss, Jena, Germany). One ear was treated with a single drop (100 µL) of the RhB-loaded optimal formula, and the other ear was treated with the RhB aqueous solution. After 6 h, the animals were euthanized, and their tympanic membranes (TMs) were cleaned and preserved. RhB fluorescence was measured using argon and helium–neon lasers at 485 and 595 nm, respectively. The images were processed and analyzed with LSM software (Carl Zeiss Microimaging, Jena, Germany) (Ahmed et al. 2025b; Younes & Habib 2022).

#### Statistical analysis

A 2^3^ full factorial experimental design was implemented to systematically investigate the effect of formulation variables on key response parameters. Each experimental run was performed in triplicate, and data were expressed as mean ± standard deviation (SD). To determine the statistical significance of differences among groups, one-way analysis of variance (ANOVA) was utilized. This method allowed for the comparison of means across multiple formulation groups to assess whether observed differences were statistically meaningful. A *p* value less than 0.05 was considered significant. When significant differences were found, the least significant difference (LSD) post hoc test was applied to pinpoint specific group comparisons showing statistically relevant variation (Ahmed et al. 2024b, a).

## Results and discussions

### Factorial design analysis

Statistical design methodologies were employed to systematically assess the influence of individual formulation variables on the characteristics of the delivery system. Each factor was evaluated using different model orders, allowing for independent analysis and model fitting. Numerical optimization, based on polynomial regression models, was utilized to identify the most suitable formulation, ensuring high predictive accuracy. Each response variable was analyzed individually, and the best-fitting model was selected accordingly. As presented in Table 3, the values for adjusted *R*^2^, predicted *R*^2^, and adequate precision demonstrated good alignment, confirming the reliability of the models. Notably, most responses exhibited adequate precision values exceeding the threshold of 4, indicating sufficient signal-to-noise ratios (Habib et al. 2018).

**Table 3:** Model analysis for studied responses

Response	*R*^2^	Adjusted *R*^2^	Predicated *R*^2^	Adequate precision	Significant factors
EE%	0.9936	0.9888	0.9743	37.807	A, B, C
PS (nm)	0.9849	0.9735	0.9395	24.893	A, B, C
ZP (mV)	0.9624	0.9343	0.8497	15.658	B, C

*EE%*, percent entrapment efficiency; *PS*, particle size; *ZP*, zeta potential

#### Evaluation of factors influencing EE%

**Fig. 1:**
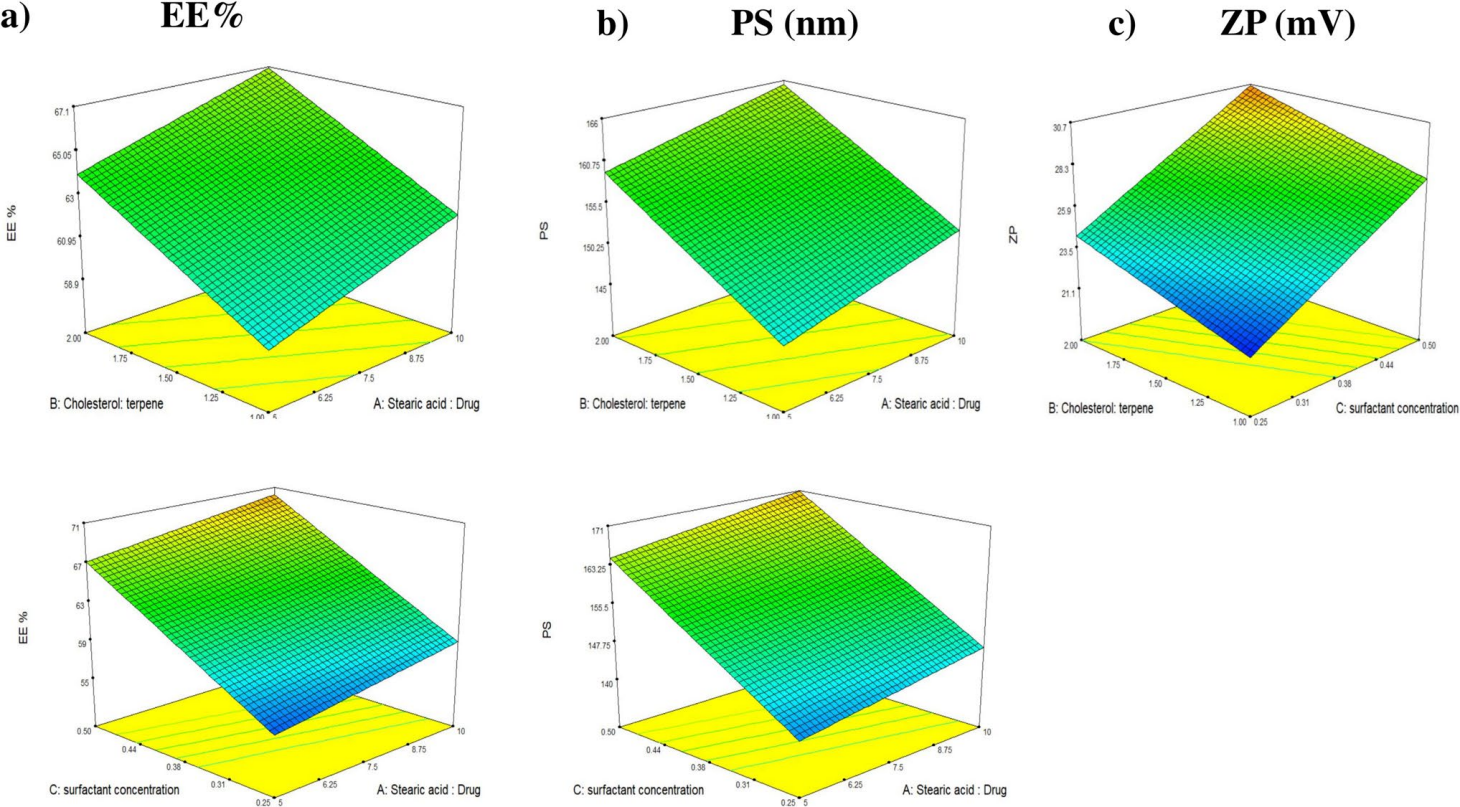
Response 3D plots for the effect of factor (A) stearic acid:drug ratio, factor (B) cholesterol:terpene (fenchone) ratio, and factor (C) surfactant concentration (%) on **a** EE%, **b** PS, and **c** ZP

Achieving effective treatment of otic infections requires the efficient delivery of levofloxacin (LFX) to the infection site, underscoring the importance of a high entrapment efficiency (EE%) (Younes et al. 2024). The developed formulations exhibited EE% values ranging from 53.87 ± 0.72 to 73.39 ± 7.49%, as shown in Table 2**.** Analysis of variance (ANOVA) revealed that all three studied factors: stearic acid drug ratio (factor A), cholesterol:fenchone ratio (factor B), and surfactant concentration (factor C) had exerted a statistically significant positive effect on EE%, as illustrated in Fig. 1a. The regression equation derived from the coded variables was as follows:

EE% = 63.01 + 1.53A + 2.50B + 5.66C.

The increase in EE% can be attributed to both the individual and synergistic roles of these formulation components. Stearic acid (factor A) contributes to the formation of a less ordered, amorphous lipid matrix due to its saturated long-chain structure. This reduces the overall crystallinity of the nanoparticles, creating more space within the lipid matrix to accommodate LFX and limiting drug leakage (Ahmed et al. 2022a; Emad Eldeeb et al. 2019b). Simultaneously, an increase in the cholesterol concentration led to a notable enhancement in entrapment efficiency. This improvement can be attributed to cholesterol’s amphiphilic characteristics, which contribute to both the formation and structural reinforcement of novasomes. By increasing the lipid bilayer’s hydrophobicity and mechanical rigidity, cholesterol plays a critical role in stabilizing vesicular membranes. These findings are in line with previously reported data highlighting cholesterol’s pivotal role in improving the stability and encapsulation performance of lipid-based delivery systems (Abdelbari et al. 2021; Lens 2025). Additionally, surfactants (factor C) reduce interfacial tension and stabilize vesicle formation. Their incorporation improves the structural integrity of the lipid bilayer, allowing more drug to be entrapped and retained. The marked increase in EE% with rising surfactant concentration suggests a synergistic enhancement of encapsulation through both improved vesicle stability and increased bilayer accommodation capacity. These findings align with previous reports, including those by El-Laithy et al., who demonstrated a similar role for surfactants in enhancing the EE% of vesicular systems for transdermal drug delivery (El-Laithy et al. 2011).

#### Evaluation of variables influencing PS

Achieving an optimal particle size (PS) is critical for ensuring efficient drug retention and permeation through the tympanic membrane (TM), with smaller particles generally offering superior penetration (A. A. Abdelbary et al. 2019). As presented in Table 2, the measured PS of the developed formulations ranged from 133.90 ± 1.41 to 179.25 ± 3.61 nm. Statistical analysis using ANOVA confirmed that all three formulation variables—factor A (stearic acid:drug ratio), factor B (cholesterol:fenchone ratio), and factor C (surfactant concentration) had a significant positive influence on particle size, as illustrated in Fig. 1b. The corresponding regression equation based on coded values was as follows:

PS = 155.74 + 3.04A + 6.71B + 12.09C.

An increase in stearic acid content (factor A) was associated with larger particle sizes, likely due to its high melting point (69.3 °C), which may hinder efficient homogenization. This thermal property can lead to slower lipid phase transitions, producing particles with broader size distributions and larger diameters (Subroto et al. 2022). Cholesterol (factor B), known for its amphiphilic and bulky molecular structure, also contributed to an increase in PS. Its integration into the bilayer enhances membrane rigidity due to the alignment of hydrophilic head groups outward and lipophilic tails inward. This spatial configuration stiffens the bilayer, promoting the formation of larger and more structurally stable vesicles capable of encapsulating greater drug quantities (Emad Eldeeb et al. 2019a). Surfactant concentration (factor C) showed a linear correlation with PS. Higher surfactant levels facilitated the formation of larger vesicles, which may be attributed to increased drug loading efficiency. The surfactants likely expanded the bilayer to accommodate more LFX, thereby increasing overall vesicle size. These results are consistent with previously published studies (Albash et al. 2022b).

Collectively, these three components exhibit synergistic effects in modulating vesicle size. Stearic acid contributes to morphological structure, cholesterol enhances rigidity and entrapment potential, and surfactants stabilize the interface while promoting expansion. Together enabling the formation of vesicles optimized for effective otic delivery.

#### Evaluation of variables influencing ZP

Zeta potential (ZP) reflects the stability of the prepared system by indicating the total charge on the particles, with ZP generating repulsive forces that enhance stability. In this study, the values of ZP ranged from − 20.80 ± 0.28 to − 31.95 ± 0.21 (Table 2). The cholesterol:fenchone ratio (factor B) and surfactant concentration (factor C) showed a significant positive effect on ZP. The equation derived from the coded factors is as follows:

ZP = 25.89 + 0.94A + 1.54B + 3.19C.

Cholesterol possesses ionizable hydroxyl groups that can contribute to surface charge development upon ionization, leading to an increase in the absolute value of zeta potential (ZP). As the cholesterol content rises, the resulting enhancement in surface charge promotes stronger electrostatic repulsion between vesicles, thereby improving colloidal stability (Emad Eldeeb et al. 2019b; Younes et al. 2024). Additionally, Span being non-ionic in nature played a complementary role by lowering the interfacial tension between the vesicles and the surrounding medium. This reduction in interfacial tension limits vesicle coalescence and aggregation. The combination of increased electrostatic repulsion from cholesterol and steric stabilization from non-ionic surfactants acts synergistically to enhance the physical stability of the vesicular system.

#### Evaluation of variables influencing PDI

The poly-dispersity index (PDI) measures particle distribution, with a value of 0 indicating a highly uniform system and 1 indicating a highly diverse system (Teama et al. 2025; Nemr et al. 2022). In this study, the PDI values of the LFX-loaded formulations ranged from 0.12 ± 0.02 to 0.42 ± 0.07 (Table 2), indicating acceptable homogeneity. ANOVA analysis showed that the studied variables had no significant impact on PDI, leading to its exclusion from the optimization criteria.

### Choosing the optimum formula

The optimal formula composition, determined through numerical optimization, consisted of a stearic acid to drug ratio of 10, a cholesterol to fenchone ratio of 2, and a surfactant concentration of 0.5%, with a desirability of 0.964. Table 4 shows the percentage differences between observed and predicted outcomes for EE%, PS, and ZP, confirming the optimization’s validity. The percentage deviation for all dependent variables was less than 5%. More experiments were conducted with this formulation (Helal et al. 2024).

**Table 4:** Characterization of the optimum formula

Response	Y1	Y2	Y4
	**EE%**	**PS (nm)**	**ZP (mV)**
**Observed value**	73.39	179.25	− 31.95
**Predicated value**	72.70	177.59	− 31.55
**% deviation (absolute)**	0.95	0.94	1.27

*EE%,* percent entrapment efficiency; *PS*, particle size; *ZP*, zeta potential

### In vitro characterization of the optimum formula

#### FTIR

**Fig. 2:**
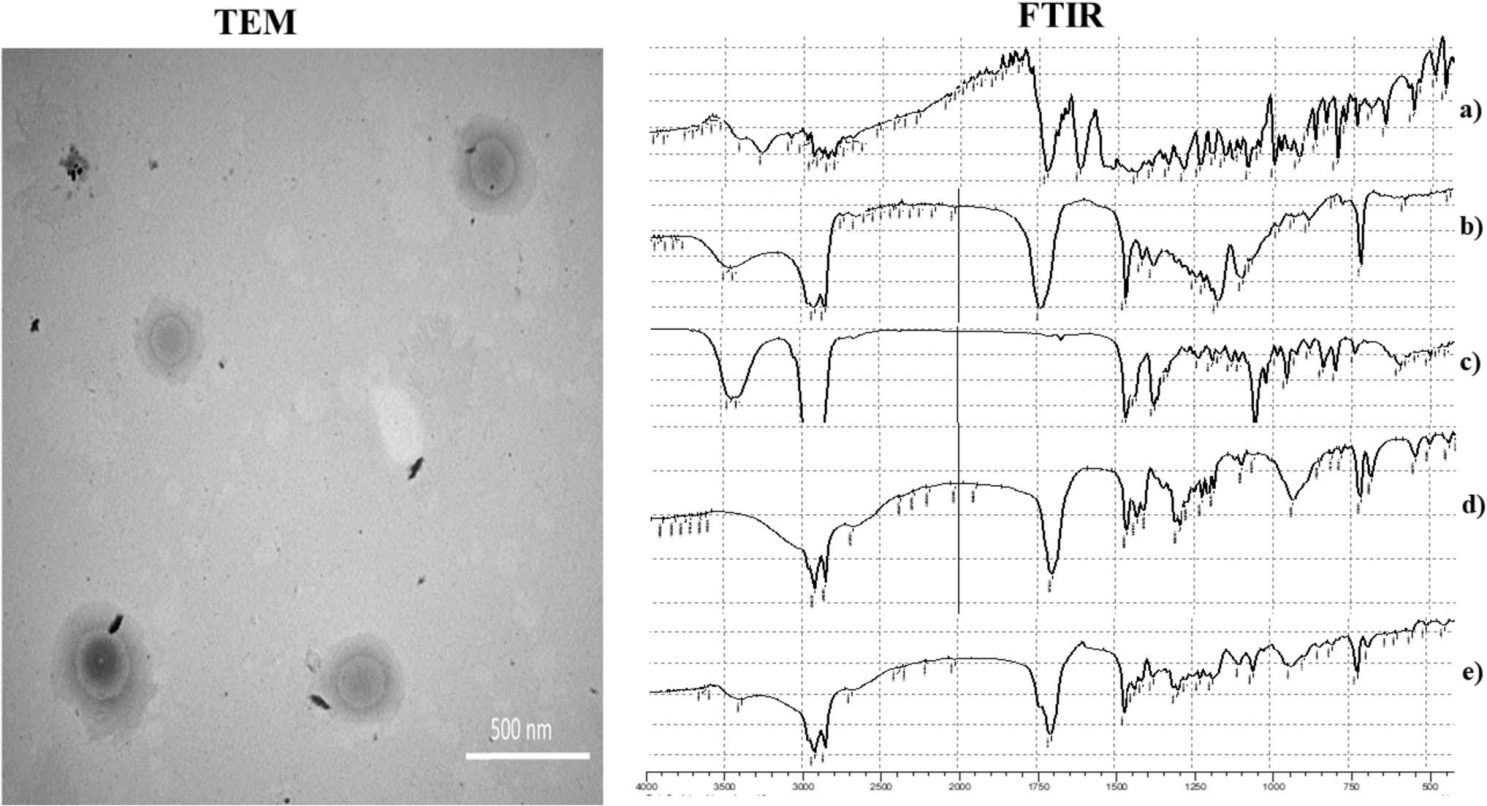
FTIR spectra of pure **a** LFX, **b** span 65, **c** cholesterol, **d** stearic acid, and **e** lyophilized optimum formula. In addition to TEM of the optimum formula

Figure 2 shows the FTIR spectrum of pure LFX, Span 65, cholesterol, stearic acid, and the lyophilized optimal formula. FTIR peaks for LFX are observed at 1700, 1600, and 3350 cm⁻^1^, corresponding to alkenyl stretching, aliphatic bending, and amino stretching, respectively (T. Zhang et al. 2022). The characteristic peaks of Span 65 and cholesterol appear around 3450, 2900, and 1700 cm⁻^1^, corresponding to hydroxyl, aliphatic CH, and carbonyl groups, respectively (El-Gazayerly et al. 2014). The broad peak of stearic acid around 2500–3300 cm⁻^1^ corresponds to the carboxylic acid group, while peaks at 2900 and 1700 cm⁻^1^ are attributed to aliphatic CH and C = O groups (Zhu et al. 2016). FTIR analysis confirmed the successful encapsulation of LFX, as its characteristic peaks were absent in the optimal formulation (Ahmed et al. 2022a).

#### Transmission electron microscopy

The transmission electron microscopy (TEM) image of the optimized formulation (Fig. 2) reveals uniformly distributed, spherical Novasomes with no signs of aggregation. The observed size range closely correlates with the dynamic light scattering results obtained from the Malvern Zetasizer, confirming consistency across characterization methods. This colloidal stability can be attributed to a synergistic interplay between steric hindrance provided by the incorporated non-ionic surfactants and electrostatic repulsion arising from the negatively charged vesicle surfaces. Together, these mechanisms effectively prevent vesicle coalescence and promote structural integrity. These findings are in accordance with previously reported studies that demonstrated similar stabilization behavior in vesicular systems (R. Albash et al. 2022a, b; Sakr et al. 2023).

#### In vitro drug release profiling and kinetic modeling

**Fig. 3:**
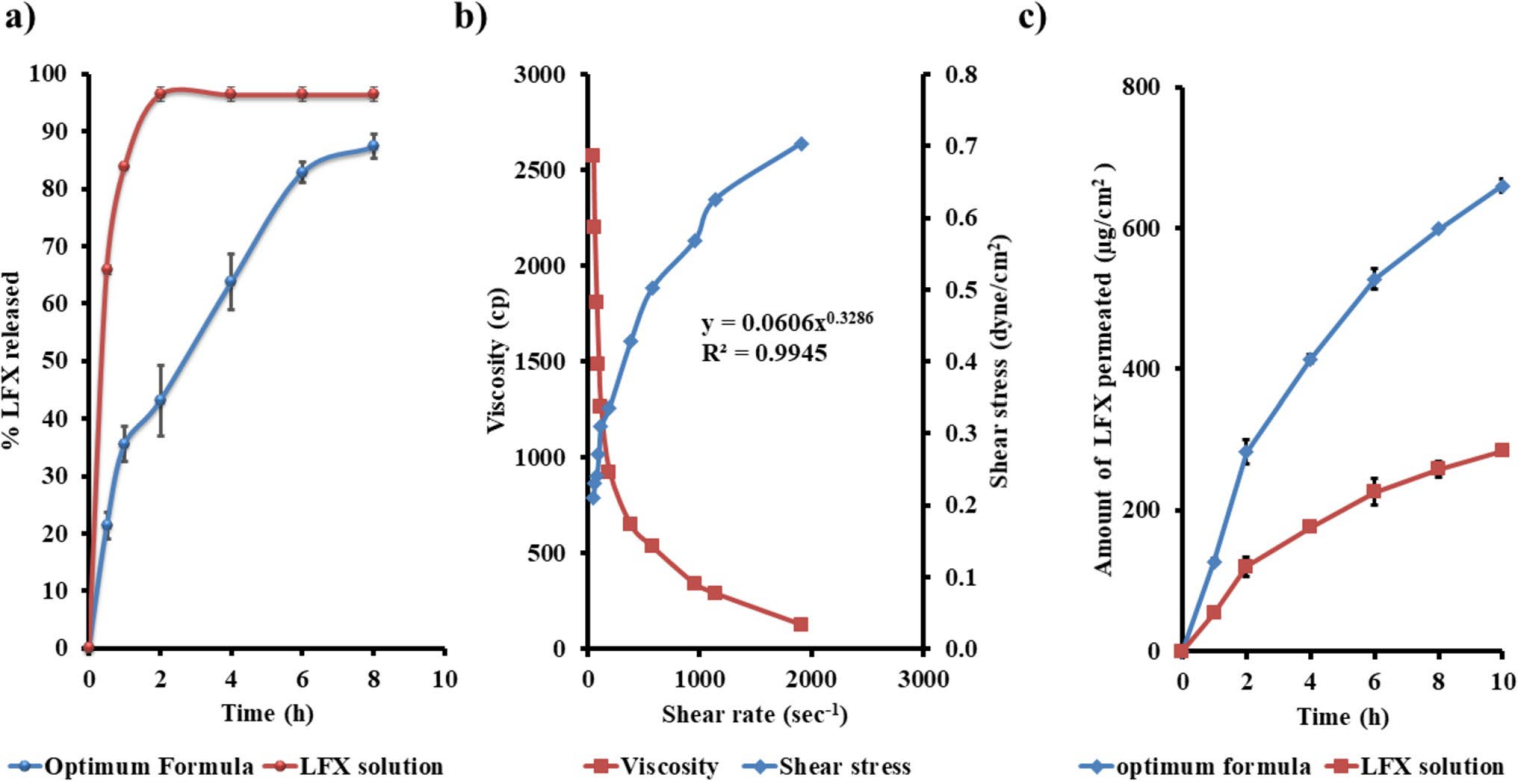
**a** In vitro release profile of the optimum formula compared to LFX solution. **b** Rheological characterization of the optimum formula. **c** Ex vivo trans-tympanic permeation profile of the optimum formula compared to LFX solution

Although drug release was not included as a dependent variable in the optimization design, it was thoroughly evaluated for the optimized formulation. The focus of the factorial design was on parameters directly influenced by formulation variables. A subsequent release study confirmed a desirable sustained release profile, supporting the formulation’s suitability for trans-tympanic delivery. The optimized formulation exhibited a biphasic release profile, characterized by an initial rapid release followed by a sustained release phase, and demonstrated a significantly slower drug release compared to the plain LFX solution (Fig. 3a). During the initial phase, approximately 35% of LFX was released within the first 2 h, likely due to the desorption of surface-associated, un-encapsulated drug molecules (C. Yousry et al. 2020). This was followed by a prolonged release phase, attributed to the strong affinity of LFX for the lipophilic core of the novasomes. The sustained release behavior is a result of the synergistic interaction between Span 65 and cholesterol. Span 65, a non-ionic surfactant, promotes vesicle stability by reducing permeability, while cholesterol enhances membrane rigidity by reinforcing the lipid bilayer structure. Together, these components reduce the formation of aqueous channels and limit drug diffusion, thereby prolonging release. Kinetic modeling of the release data confirmed that the mechanism conformed to the Higuchi diffusion model, indicating that drug release was governed primarily by diffusion through the lipid matrix.

#### Rheological studies

The study found that the optimal formulation displayed shear-thinning behavior, as shown by viscosity drop with increasing shear rate (Fig. 3b). A power law model indicated non-Newtonian and shear-thinning properties, with a flow index (*n*) significantly less than one (*n* = 3286). Additionally, the Carreau model showed the highest *R*^2^ value, indicating a pseudo-plastic flow behavior (Ahmed et al. 2025d). This flow behavior is advantageous for otic formulations, as it allows for easier insertion into the ear canal with reduced viscosity at higher shear rates (e.g., during shaking). At low shear rates, it maintains its structure, which enhances stability and improves retention (A. A. Nemr et al. 2021). Reduced viscosity is due to the alignment of molecules in the direction of flow under shear stress. This results in a decrease in apparent molecular weight and the concentration of dispersed molecules, leading to the release of some retained water (A. M. Fahmy et al. 2018).

#### Storage effect

No aggregation or changes in physical appearance were observed in the stored formula. Statistical analysis of entrapment efficiency (EE%), particle size (PS), and zeta potential (ZP) before and after storage, shown in Table 5, indicated no significant differences. A similarity factor value of 77.74 further confirmed the comparable in vitro release patterns. The optimal formula’s exceptional stability is likely due to the strong negative charge of hydroxyl groups which ionized in cholesterol and the stabilizing effects of surfactants which help prevent agglomeration (Ahmed et al. 2024b, a; Sayed et al. 2020).

**Table 5:** Effect of short- term stability on the optimum formula

Parameter	Fresh	Storage for 3 months at 4–8 °C	
		**Value**	Probability (*p*)*
**EE%**	73.39 ± 7.49	70.24 ± 1.36	0.617
**PS**	179.2 5 ± 3.61	172.90 ± 6.51	0.351
**ZP**	− 31.95 ± 0.21	− 32.90 ± 1.13	0.363

*EE%,* percent entrapment efficiency; *PDI*, poly-dispersity index; *PS*, particle size; *ZP*, zeta potential

^*^One-way ANOVA analysis to compare between the freshly prepared and the stored optimum formula

### Ex vivo evaluation of trans-tympanic drug permeation

Figure 3 c represents the permeation profile of the optimal formulation and the LFX solution. After 10 h, optimal formula showed a noticeably higher cumulative amount of LFX permeated per unit area (659.95 ± 9.57 µg/cm^2^) and improved flux (65.99 ± 0.96 µg/cm^2^/h), compared to the LFX solution (284.55 ± 6.36 µg/cm^2^ and 28.45 ± 0.64 µg/cm^2^/h). The enhancement ratio of 2.32 indicates the formula’s effectiveness. Decreasing particle size of the optimal formula likely improved its residence time and passage through the tympanic membrane, while cholesterol enhanced diffusion, contributing to the superior results (Emad Eldeeb et al. 2019b). Span 65 reduced surface tension between the optimal formulation and the tympanic membrane, and also minimizing strong epithelial interactions. Span 65 may also enhance drug transmission through the tympanic membrane by improving solubility, dispersion, disrupting lipid bilayers, and promoting penetration (Ahmed et al. 2022b). Furthermore, fenchone temporarily modify the intercellular lipid architecture, reducing the rigidity of tympanic membrane. This disruption facilitates enhanced diffusion of LFX across the intact membrane (Ahmed et al. 2025e; Ibrahim et al. 2024b).

### Microbiological assays

#### Determination of MIC and MBC

The antibacterial activity of the optimized formula was compared to plain LFX solution to confirm the overall enhancement provided by the new delivery system. Since fenchone is a fixed component of the optimized formula, its effect was not separated. The goal was to assess the combined action of all ingredients as a single therapeutic system. The optimal formula showed significantly lower minimum inhibitory concentrations (MIC) against *Staphylococcus aureus* USA300 (0.977 μg/mL) and *Pseudomonas aeruginosa* PAO1 (7.8 μg/mL) in comparison with the drug solution (15.6 and 125 μg/mL, respectively). Additionally, both formulations exhibited bactericidal activity after 24 h of incubation at 35 °C ± 2. The optimal formula demonstrated significantly lower minimum bactericidal concentrations (MBC) against *Staphylococcus aureus* USA300 (3.9 μg/mL) and *Pseudomonas aeruginosa* PAO1 (31.25 μg/mL), compared to the drug solution (62.5 and 250 μg/mL, respectively). These results suggest that the optimal formula exhibits enhanced antibacterial activity.

#### The biofilm inhibition activity

**Fig. 4:**
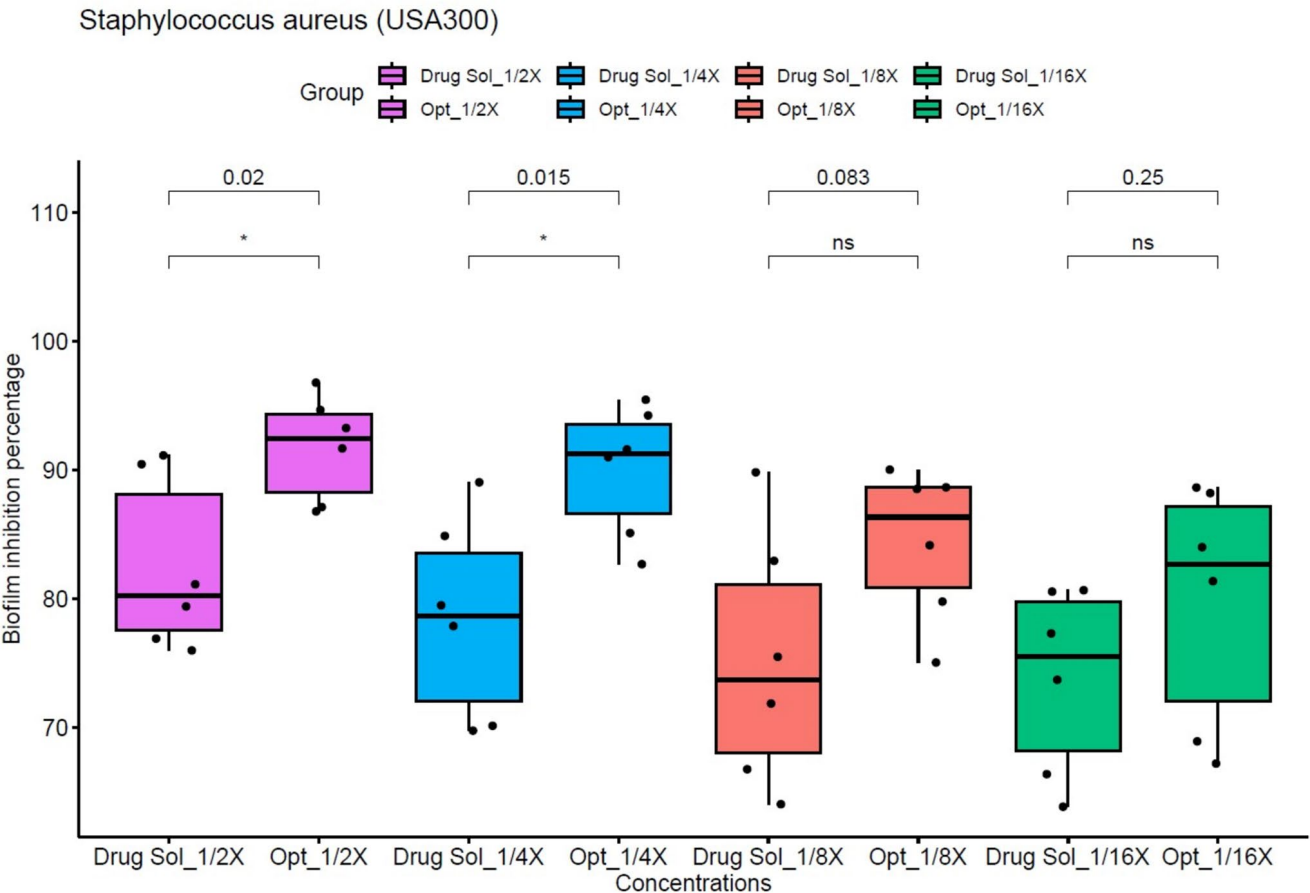
The biofilm inhibition activity against *Staphylococcus aureus* USA 300 standard strain. Biofilm inhibitory effect of different concentrations (^1^/_16_, ^1^/_8_, ¼, ½, X, where X is the calculated MIC) of the optimized formula (Opt) and the drug solution (Drug Sol). Optimized formula concentrations are as follows: ^1^/_16_ X= 0.06 μg/mL, ^1^/_8_ X = 0.122 μg/mL, ¼ X = 0.244 μg/mL, ½ X = 0.488 μg/mL. Drug solution concentrations are as follows: ^1^/_16_ X = 0.975 μg/mL, ^1^/_8_ X = 1.95 μg/mL, ^1^/_4_ X = 3.9 μg /mL, ½ X = 7.8 μg/mL

**Fig. 5:**
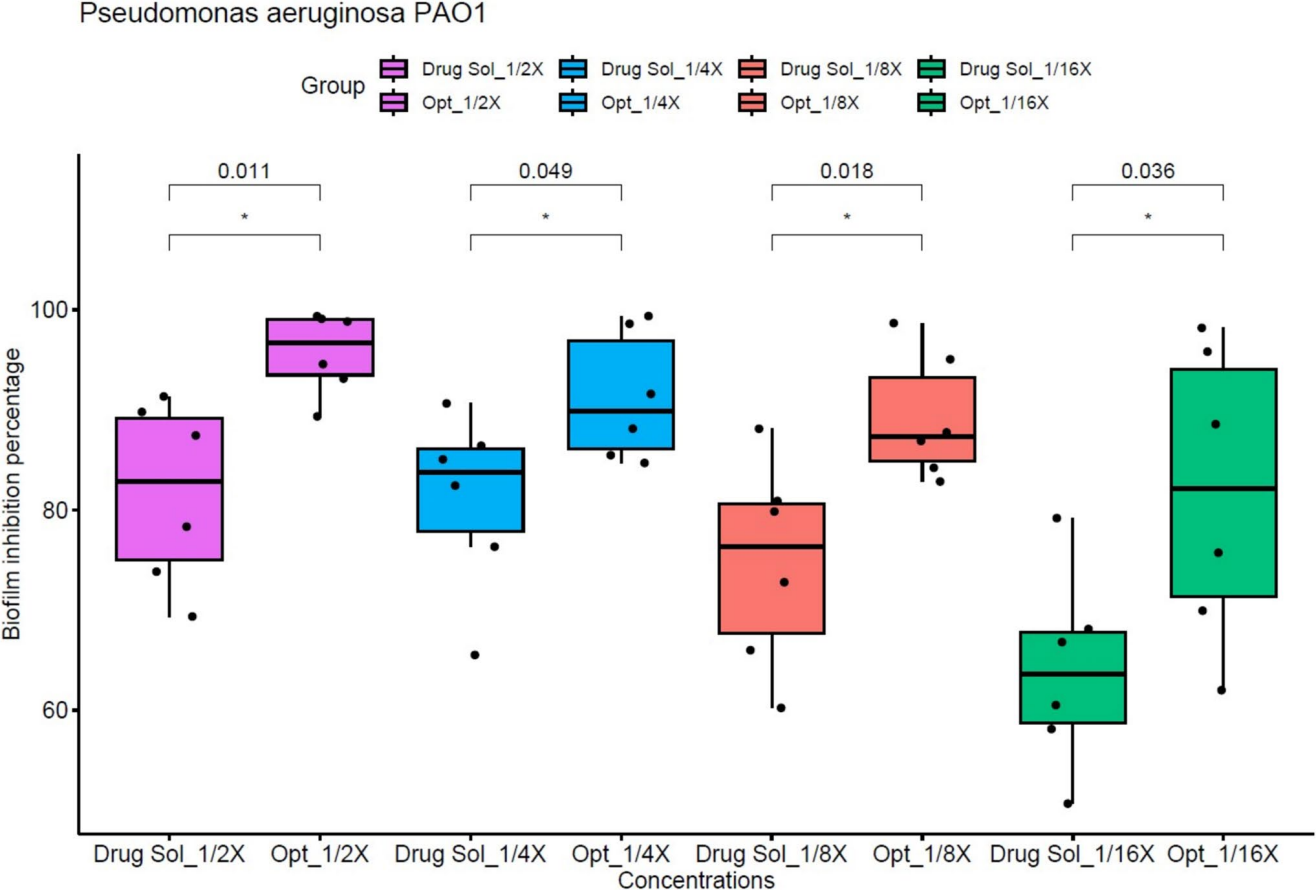
The biofilm inhibition activity against *Pseudomonas aeruginosa* PAO1 standard strain. Biofilm inhibitory effect of different concentrations (^1^/_16_, ^1^/_8_, ¼, ½, X, where X is the calculated MIC) of the optimized formula (Opt) and the drug solution (Drug Sol). Optimized formula concentrations are as follows: ^1^/_16_ X= 0.487 μg/mL, ^1^/_8_ X = 0.975 μg/mL, ^1^/_4_ X = 1.95 μg/mL, ^1^/_8_ X = 3.9 μg/mL. Drug solution concentrations are as follows: ^1^/_16_ X = 7.8 μg/mL, ^1^/_8_ X = 15.6 μg/mL, ^1^/_4_ X = 31.25 μg /mL, ½ X = 62.5 μg/mL

The microbial biofilm inhibition activity of both the drug solution and the optimal formula was assessed at concentrations equal to ^1^/_16_, ^1^/_8_, ^1^/_4_ and ^1^/_2_ X, where X represents the MIC determined for each preparation. The optimal formula exhibited a much stronger biofilm inhibition activity compared to the drug solution against *Staphylococcus aureus* USA300 at ^1^/_2_ and ^1^/_4_ of the MIC concentration (Student’s *t* test, *p* < 0.05, Fig. 4). Additionally, at all tested concentrations, the optimal formula demonstrated noticeably higher inhibition of biofilm activity than the drug solution against *Pseudomonas aeruginosa* PAO1 (Student’s* t* test, *p* < 0.05), as presented in Fig. 5. Statistical analysis was performed using R version 4.1.2 and visualized in RStudio. The enhanced efficacy of the optimized formulation can be attributed to its nanoscale particle size, which facilitate precise interaction with bacterial cells, thereby improving antimicrobial performance. Moreover, the incorporated surfactants might interfere with biofilm development, disrupting its structural integrity. In parallel, fenchone has been reported to contribute to membrane destabilization of bacterial cells, which could promote leakage of essential intracellular contents and ultimately lead to cell death (Allegrone et al. 2021).

### In vivo analysis

#### Histopathological examination

**Fig. 6:**
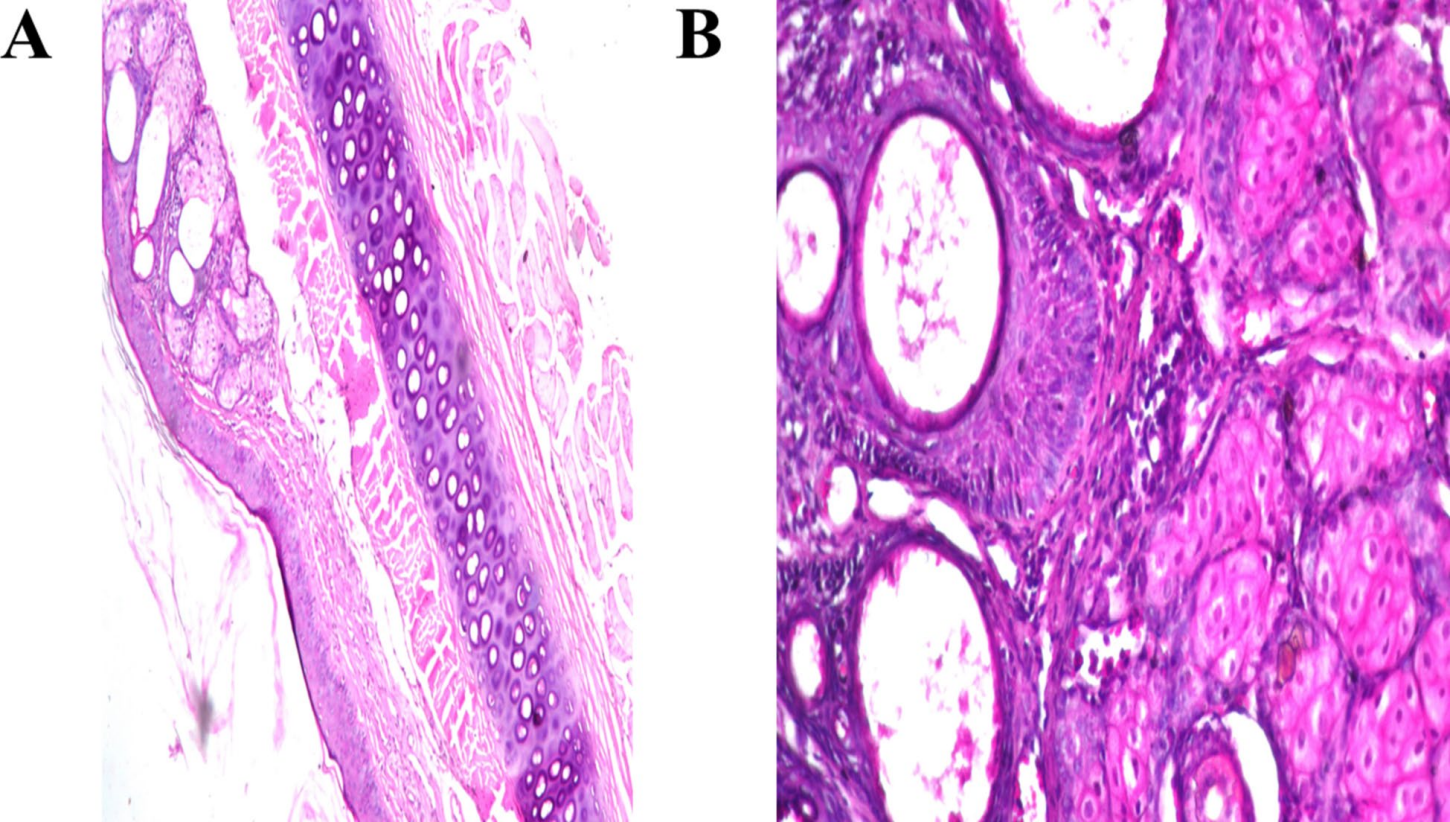
Histopathological sections of rabbits’ tympanic membrane after instillation of **A** normal saline solution (negative control) and **B** optimum formula

The tympanic membranes (TMs) of rabbits exposed to the negative control (normal saline) showed normal histology with no signs of irritation or inflammation, as demonstrated in Fig. 6A. In contrast, TMs treated with the optimal formula maintained a normal structure with no signs of irritation or inflammation, as illustrated in Fig. 6B**.** These findings confirm the safe use of novasomes for otic applications. The selection of components and their concentrations in the formula were thoughtfully made to adhere to safety standards and minimize potential adverse effects. This focus on safety highlights the potential of novasomes as a reliable and effective delivery system for otic treatments (A. A. Nemr et al. 2023).

#### In vivo permeation

**Fig. 7:**
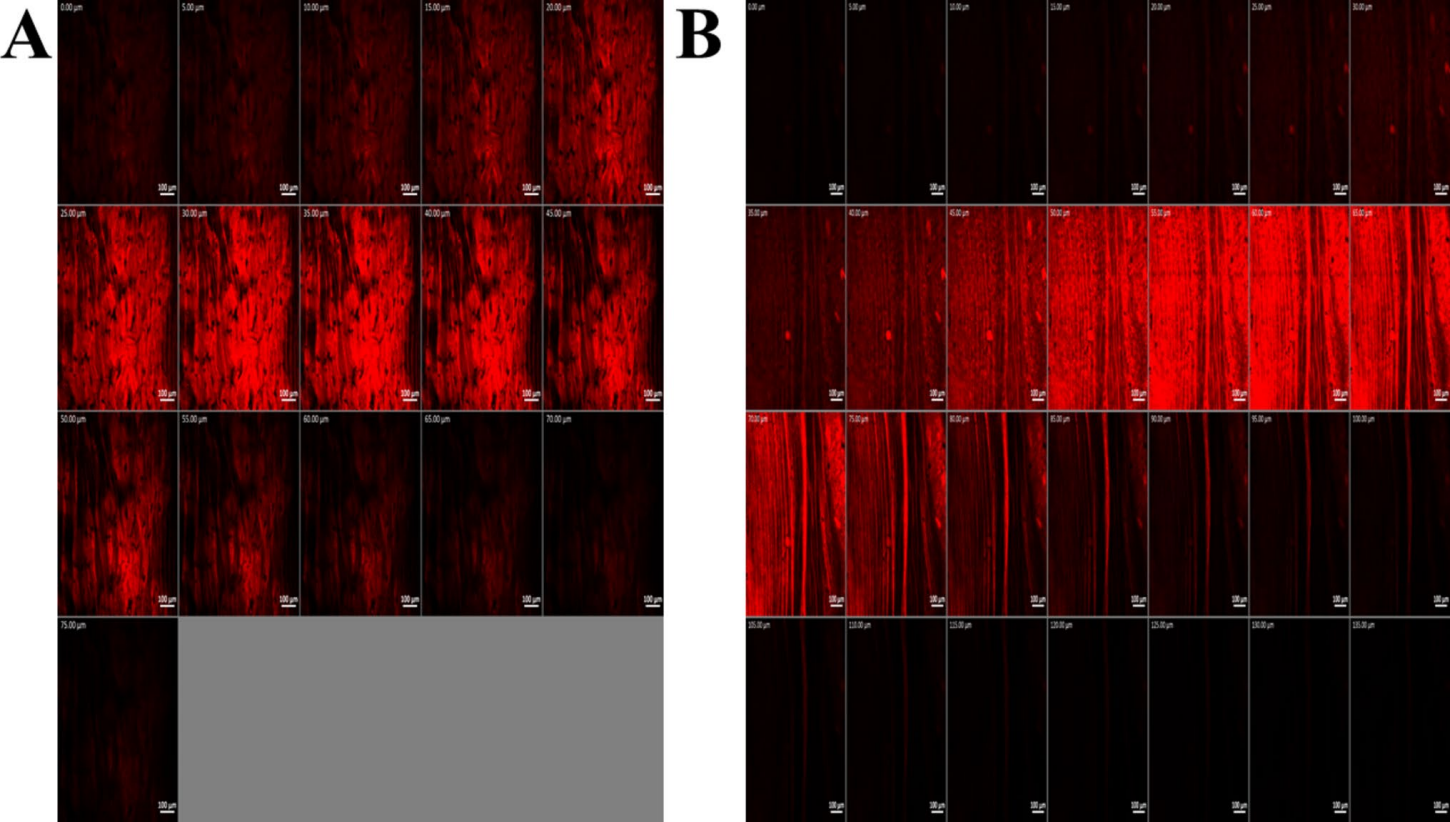
Confocal laser scanning micrographs of rabbits’ tympanic membrane after instillation of **A** RhB aqueous solution and **B** RhB-loaded optimum formula

The in vivo uptake study using confocal laser scanning microscopy (CLSM) confirmed the enhanced performance of the optimal novasome formulation. It achieved 135-μm permeation depth, significantly surpassing the aqueous solution’s 75 μm, as illustrated in Fig. 7. The optimal formulation also demonstrated a 1.8-fold increase in RhB permeation due to its unique design, improving drug delivery across biological membranes. These results highlight novasomes’ potential to effectively penetrate the tympanic membrane, enhancing their therapeutic application for otitis media treatment.

## Limitations of the Study

While the present study demonstrates the potential of fenchone-enriched novasomes as a promising non-invasive otic drug delivery system, some limitations should be acknowledged. First, the in vivo experiments were conducted on animal models, which, although valuable, may not fully replicate the complexity of human middle ear physiology and pathology. Second, the long-term safety and pharmacokinetics of the optimized formulation were not assessed beyond the short-term exposure period. Third, although the formulation exhibited enhanced antibacterial and anti-biofilm activities, resistance development and spectrum specificity were not investigated in detail.

## Conclusions and future prospective

In this study, a novel fenchone-enriched novasomal delivery system was successfully developed using the ethanol injection method to enhance the trans-tympanic delivery of levofloxacin (LFX) for the treatment of otitis media. The optimized formulation exhibited distinct physicochemical advantages, including high entrapment efficiency, nanoscale particle size, a stable zeta potential, and a biphasic sustained drug release profile. Fourier-transform infrared spectroscopy (FTIR) confirmed successful drug encapsulation, while transmission electron microscopy (TEM) revealed uniform, spherical vesicles with excellent colloidal stability. Ex vivo studies demonstrated significantly improved permeation through the tympanic membrane compared to conventional LFX solution. Moreover, the optimized novasomes showed superior antibacterial and anti-biofilm activity, particularly against AOM-associated pathogens, and were proven to be histologically safe for otic application. These findings collectively support the innovative potential of fenchone-loaded novasomes as a non-invasive, targeted therapy for middle ear infections, minimizing systemic side effects and enhancing local bioavailability.

Based on these findings, future research should prioritize clinical trials to validate the efficacy and safety of this system in human subjects, along with pharmacokinetic profiling, long-term safety evaluation, and regulatory scalability assessments to support clinical translation and commercialization. Incorporating this delivery system into pediatric treatment protocols may enhance patient compliance and therapeutic outcomes. Additionally, expanding the platform to encapsulate other therapeutic agents could establish novasomes as a versatile nanocarrier for otic and broader mucosal drug delivery applications.

## Data Availability

All source data for this work (or generated in this study) are available upon reasonable request.

## Declarations

Ethical statements.

## References

[CR1] Abdelbari MA, El-Mancy SS, Elshafeey AH, Abdelbary AA (2021) Implementing spanlastics for improving the ocular delivery of clotrimazole: in vitro characterization, ex vivo permeability, microbiological assessment and in vivo safety study. Int J Nanomedicine 16:6249–6261. 10.2147/IJN.S319348 34531656 10.2147/IJN.S319348 PMC8439980

[CR2] Abdelbary GA, Amin MM, Abdelmoteleb M (2016) Novel mixed hydrotropic solubilization of zaleplon: formulation of oral tablets and in-vivo neuropharmacological characterization by monitoring plasma GABA level. J Drug Deliv Sci Technol 33:98–113. 10.1016/j.jddst.2016.03.014

[CR3] Abdelbary AA, Abd-Elsalam WH, Al-Mahallawi AM (2019) Fabrication of levofloxacin polyethylene glycol decorated nanoliposomes for enhanced management of acute otitis media: statistical optimization, trans-tympanic permeation and in vivo evaluation. Int J Pharm 559:201–209. 10.1016/j.ijpharm.2019.01.037 30684597 10.1016/j.ijpharm.2019.01.037

[CR4] Abdelhakeem E, Nemr AA, Rashed HM, Selim AA, Essa BM, Hegazy D (2025) Revitalizing itraconazole: unleashing its anticancer potential through oral nanosystems for liver targeting and biodistribution profiling in an animal model using radiolabeling technique. J Drug Deliv Sci Technol 104:106463. 10.1016/j.jddst.2024.106463

[CR5] Adel IM, ElMeligy MF, Abdelrahim MEA, Maged A, Abdelkhalek AA, Abdelmoteleb AMM, Elkasabgy NA (2021) Design and characterization of spray-dried proliposomes for the pulmonary delivery of curcumin. Int J Nanomedicine 16:2667–2687. 10.2147/IJN.S306831 33854314 10.2147/IJN.S306831 PMC8039018

[CR6] Adwan S, Abu-Dahab R, Al-Bakri AG, Sallam A (2015) Glyceryl monooleate-based otic delivery system of ofloxacin: release profile and bactericidal activity. Pharm Dev Technol 20(3):361–366. 10.3109/10837450.2013.871030 24392877 10.3109/10837450.2013.871030

[CR7] Ahmed S, Amin MM, El-Korany SM, Sayed S (2022a) Corneal targeted fenticonazole nitrate-loaded novasomes for the management of ocular candidiasis: preparation, in vitro characterization, ex vivo and in vivo assessments. Drug Deliv 29(1):2428–2441. 10.1080/10717544.2022.2103600 35880688 10.1080/10717544.2022.2103600 PMC9341384

[CR8] Ahmed S, Amin MM, El-Korany SM, Sayed S (2022b) Pronounced capping effect of olaminosomes as nanostructured platforms in ocular candidiasis management. Drug Deliv 29(1):2945–2958. 10.1080/10717544.2022.2120926 36073061 10.1080/10717544.2022.2120926 PMC9848414

[CR9] Ahmed S, Attia H, Saher O, Fahmy AM (2024a) Augmented glycerosomes as a promising approach against fungal ear infection: optimization and microbiological, ex vivo and in vivo assessments. Int J Pharm: X 8:100295. 10.1016/j.ijpx.2024.100295 39525529 10.1016/j.ijpx.2024.100295 PMC11543555

[CR10] Ahmed S, Aziz DE, Sadek MA, Tawfik MA (2024b) Capped flexosomes for prominent anti-inflammatory activity: development, optimization, and ex vivo and in vivo assessments. Drug Deliv Transl Res 14(9):2474–2487. 10.1007/s13346-024-01522-z 38315262 10.1007/s13346-024-01522-z PMC11525274

[CR11] Ahmed S, Farag MM, Attia H, Balkhi B, Adel IM, Nemr AA (2025a) Exploring the potential of antifungal-loaded proniosomes to consolidate corneal permeation in fungal keratitis: a comprehensive investigation from laboratory characterization to microbiological evaluation. International Journal of Pharmaceutics: X 9:100322. 10.1016/j.ijpx.2025.100322 40094144 10.1016/j.ijpx.2025.100322 PMC11909449

[CR12] Ahmed S, Farag MM, Attia H, Balkhi B, Adel IM, Nemr AA (2025b) Terconazole loaded edge-activated hybrid elastosome for revamped corneal permeation in ocular mycosis: in-vitro characterization, statistical optimization, microbiological assessment, and in-vivo evaluation. Int J Pharm: X 9:100333. 10.1016/j.ijpx.2025.100333 40292341 10.1016/j.ijpx.2025.100333 PMC12023791

[CR13] Ahmed S, Farag MM, Sadek MA, Aziz DE (2025c) Transdermal application of diacerin loaded-terpene enriched invasomes: an approach to augment anti-edema and nociception inhibition activity. J Liposome Res 35(1):1–14. 10.1080/08982104.2024.2382974 39074044 10.1080/08982104.2024.2382974

[CR14] Ahmed S, Mehana D, Attia H, El-Ashmoony MM (2025d) Engineered terconazole-loaded flexogel for targeted vaginal delivery: in-depth analysis using in-vitro, microbiological, ex-vivo and in-vivo methodologies. J Drug Deliv Sci Technol 111:107192. 10.1016/j.jddst.2025.107192

[CR15] Ahmed S, Saher O, Attia H, Fahmy AM, Adel IM (2025e) Development and characterization of fenticonazole nitrate-loaded cubogel for the management of vaginal candidiasis. International Journal of Pharmaceutics: X 10:100355. 10.1016/j.ijpx.2025.100355 40703664 10.1016/j.ijpx.2025.100355 PMC12284559

[CR16] Ahmed S, Ibrahim MM, Balkhi B, Attia H, Aziz DE (2025f) From bench to biology: Unraveling the efficiency of novel brijosomes for transtympanic drug delivery. J Drug Deliv Sci Tec 114:107493. 10.1016/j.jddst.2025.107493

[CR17] Albash R, Al-Mahallawi AM, Hassan M, Alaa-Eldin AA (2021) Development and optimization of terpene-enriched vesicles (Terpesomes) for effective ocular delivery of fenticonazole nitrate: in vitro characterization and in vivo assessment. Int J Nanomedicine 16:609–621. 10.2147/IJN.S274290 33531804 10.2147/IJN.S274290 PMC7847387

[CR18] Albash R, El-Dahmy RM, Hamed MIA, Darwish KM, Alahdal AM, Kassem AB, Fahmy AM (2022a) Repurposing levocetirizine hydrochloride loaded into cationic ceramide/phospholipid composite (CCPCs) for management of alopecia: central composite design optimization, in- silico and in-vivo studies. Drug Deliv 29(1):2784–2795. 10.1080/10717544.2022.2108939 36047012 10.1080/10717544.2022.2108939 PMC9448385

[CR19] Albash R, Ragaie MH, Hassab MAE, El-Haggar R, Eldehna WM, Al-Rashood ST, Mosallam S (2022b) Fenticonazole nitrate loaded trans-novasomes for effective management of tinea corporis: design characterization, in silico study, and exploratory clinical appraisal. Drug Deliv 29(1):1100–1111. 10.1080/10717544.2022.2057619 35373684 10.1080/10717544.2022.2057619 PMC8986243

[CR20] Albash R, Abdelbari MA, Elbesh RM, Khaleel EF, Badi RM, Eldehna WM, Mosallam S (2024) Sonophoresis mediated diffusion of caffeine loaded Transcutol(R) enriched cerosomes for topical management of cellulite. Eur J Pharm Sci 201:106875. 10.1016/j.ejps.2024.106875 39121922 10.1016/j.ejps.2024.106875

[CR21] Albash R, Ali SK, Abdelmonem R, Agiba AM, Aldhahri R, Saleh A, Abdellatif MM (2025a) Electrospun nanofiber-scaffold-loaded levocetirizine dihydrochloride cerosomes for combined management of atopic dermatitis and methicillin-resistant *Staphylococcus aureus* (MRSA) skin infection: in vitro and in vivo studies. Pharmaceuticals (Basel). 10.3390/ph18050633 40430454 10.3390/ph18050633 PMC12114773

[CR22] Albash R, Fahmy AM, Shamsel-Din HA, Ibrahim AB, Bogari HA, Malatani RT, Mosallam S (2025b) Intranasal propranolol hydrochloride-loaded PLGA-lipid hybrid nanoparticles for brain targeting: optimization and biodistribution study by radiobiological evaluation. Eur J Pharm Sci 208:107061. 10.1016/j.ejps.2025.107061 40057137 10.1016/j.ejps.2025.107061

[CR23] Albash R, Yousry C, El Hassab MA, Eldehna WM, Alaa-Eldin AA (2025c) Investigation of moxifloxacin-loaded terpenes enriched cationic cerosomes (TECs) as an adjunct pulmonary therapy for COVID-19: in-silico study; D-optimal optimization; aerodynamic simulation assessment and cytotoxic evaluation. J Drug Deliv Sci Technol 106:106683. 10.1016/j.jddst.2025.106683

[CR24] Allegrone G, Ceresa C, Rinaldi M, Fracchia L (2021) Diverse effects of natural and synthetic surfactants on the inhibition of *Staphylococcus aureus* biofilm. Pharmaceutics. 10.3390/pharmaceutics13081172 34452132 10.3390/pharmaceutics13081172 PMC8402037

[CR25] Al-Mahallawi AM, Khowessah OM, Shoukri RA (2014) Nano-transfersomal ciprofloxacin loaded vesicles for non-invasive trans-tympanic ototopical delivery: in-vitro optimization, ex-vivo permeation studies, and in-vivo assessment. Int J Pharm 472(1–2):304–314. 10.1016/j.ijpharm.2014.06.041 24971692 10.1016/j.ijpharm.2014.06.041

[CR26] Al-Mahallawi AM, Khowessah OM, Shoukri RA (2017) Enhanced non invasive trans-tympanic delivery of ciprofloxacin through encapsulation into nano-spanlastic vesicles: fabrication, in-vitro characterization, and comparative ex-vivo permeation studies. Int J Pharm 522(1–2):157–164. 10.1016/j.ijpharm.2017.03.005 28279741 10.1016/j.ijpharm.2017.03.005

[CR27] El Feghaly RE, Nedved A, Katz SE, Frost HM (2023) New insights into the treatment of acute otitis media. Expert Rev Anti-Infect Ther 21(5):523–534. 10.1080/14787210.2023.2206565 37097281 10.1080/14787210.2023.2206565 PMC10231305

[CR28] El Hassab MA, Ibrahim MH, Abdel Mageed SS, Mahmoud AMA, Othman Ahmed ZS, Mosallam S, Albash R (2025) Formulation of zein nanoparticles for augmenting the anti-inflammatory activity of dexketoprofen. Front Pharmacol 16:1560585. 10.3389/fphar.2025.1560585 40667500 10.3389/fphar.2025.1560585 PMC12259680

[CR29] El-Gazayerly ON, Makhlouf AI, Soelm AM, Mohmoud MA (2014) Antioxidant and hepatoprotective effects of silymarin phytosomes compared to milk thistle extract in CCl4 induced hepatotoxicity in rats. J Microencapsul 31(1):23–30. 10.3109/02652048.2013.805836 23808477 10.3109/02652048.2013.805836

[CR30] Elgendy HA, Makky AMA, Elakkad YE, Ismail RM, Younes NF (2023) Syringeable atorvastatin loaded eugenol enriched PEGylated cubosomes in-situ gel for the intra-pocket treatment of periodontitis: statistical optimization and clinical assessment. Drug Deliv 30(1):2162159. 10.1080/10717544.2022.2162159 36604813 10.1080/10717544.2022.2162159 PMC9833412

[CR31] El-Laithy HM, Shoukry O, Mahran LG (2011) Novel sugar esters proniosomes for transdermal delivery of vinpocetine: preclinical and clinical studies. Eur J Pharm Biopharm 77(1):43–55. 10.1016/j.ejpb.2010.10.011 21056658 10.1016/j.ejpb.2010.10.011

[CR32] ElMeshad AN, Mohsen AM (2016) Enhanced corneal permeation and antimycotic activity of itraconazole against *Candida albicans* via a novel nanosystem vesicle. Drug Deliv 23(7):2115–2123. 10.3109/10717544.2014.942811 25080226 10.3109/10717544.2014.942811

[CR33] Elsadek EA, Zhang K, Hamoud YA, Mousa A, Awad A, Abdallah M, Elbeltagi A (2024) Impacts of climate change on rice yields in the Nile River Delta of Egypt: a large-scale projection analysis based on CMIP6. Agric Water Manage 292:108673. 10.1016/j.agwat.2024.108673

[CR34] Emad Eldeeb A, Salah S, Ghorab M (2019a) Formulation and evaluation of cubosomes drug delivery system for treatment of glaucoma: ex-vivo permeation and in-vivo pharmacodynamic study. J Drug Deliv Sci Technol 52:236–247. 10.1016/j.jddst.2019.04.036 10.1080/10717544.2019.1609622 PMC6534210 31090464

[CR35] Emad Eldeeb A, Salah S, Ghorab M (2019b) Proniosomal gel-derived niosomes: an approach to sustain and improve the ocular delivery of brimonidine tartrate; formulation, in-vitro characterization, and in-vivo pharmacodynamic study. Drug Deliv 26(1):509–521. 10.1080/10717544.2019.1609622 31090464 10.1080/10717544.2019.1609622 PMC6534210

[CR36] Fahmy AM, El-Setouhy DA, Ibrahim AB, Habib BA, Tayel SA, Bayoumi NA (2018) Penetration enhancer-containing spanlastics (PECSs) for transdermal delivery of haloperidol: in vitro characterization, ex vivo permeation and in vivo biodistribution studies. Drug Deliv 25(1):12–22. 10.1080/10717544.2017.1410262 29219628 10.1080/10717544.2017.1410262 PMC6058714

[CR37] Fahmy AM, Balkhi B, Sadek MA, ElBishbishy RM, Ahmed S (2025) Pegylated terpesomes of curcumin for prominent hepatoprotective activity: fabrication, optimization, biochemical analysis and in vivo evaluation. J Drug Deliv Sci Technol 108:106876. 10.1016/j.jddst.2025.106876

[CR38] Farag MM, Abdelmalak NS, El Menshawe SF, Omara AS, Hamad DS (2025) Repurposing linagliptin-loaded novasomes as a neuroprotectant for Alzheimer**’**s disease: in-vitro characterisation, statistical optimisation and ex-vivo permeation study. J Microencapsul 42(5):531–545. 10.1080/02652048.2025.2500542 40329664 10.1080/02652048.2025.2500542

[CR39] Habib BA, Sayed S, Elsayed GM (2018) Enhanced transdermal delivery of ondansetron using nanovesicular systems: fabrication, characterization, optimization and ex-vivo permeation study-Box-Cox transformation practical example. Eur J Pharm Sci 115:352–361. 10.1016/j.ejps.2018.01.044 29407555 10.1016/j.ejps.2018.01.044

[CR40] Haney EF, Trimble MJ, Hancock REW (2021) Microtiter plate assays to assess antibiofilm activity against bacteria. Nat Protoc 16(5):2615–2632. 10.1038/s41596-021-00515-3 33911258 10.1038/s41596-021-00515-3

[CR41] Hao J, Li SK (2019) Inner ear drug delivery: recent advances, challenges, and perspective. Eur J Pharm Sci 126:82–92. 10.1016/j.ejps.2018.05.020 29792920 10.1016/j.ejps.2018.05.020

[CR42] He Y, Li X (2022) The treatment effect of levofloxacin, moxifloxacin, and gatifloxacin contained in the conventional therapy regimen for pulmonary tuberculosis: systematic review and network meta-analysis. Medicine (Baltimore) 101(38):e30412. 10.1097/MD.0000000000030412 36197231 10.1097/MD.0000000000030412 PMC9509103

[CR43] Hegazy D, Fayed NM, Nour El-Din HT, Habib BA, Abdelrehim RT (2025) Optimized tazarotene transfersomes versus previously optimized tazarotene cubosomes: exploring their in vivo anti-acne activity in a *Cutibacterium acnes* inflammatory murine model. BioNanoScience 15(3):410. 10.1007/s12668-025-02009-y

[CR44] Helal AM, Yossef MM, Seif IK, Abd El-Salam M, El Demellawy MA, Abdulmalek SA, Ghareeb DA (2024) Nanostructured biloalbuminosomes loaded with berberine and berberrubine for alleviating heavy metal-induced male infertility in rats. Int J Pharm 667(Pt A):124892. 10.1016/j.ijpharm.2024.124892 39481813 10.1016/j.ijpharm.2024.124892

[CR45] Humphries RM, Ambler J, Mitchell SL, Castanheira M, Dingle T, Hindler JA (2018) CLSI methods development and standardization working group best practices for evaluation of antimicrobial susceptibility tests. J Clin Microbiol. 10.1128/JCM.01934-17 29367292 10.1128/JCM.01934-17 PMC5869819

[CR46] Ibrahim MM, Basalious EB, El-Nabarawi MA, Makhlouf AI, Sayyed ME, Ibrahim IT (2024a) Nose to brain delivery of mirtazapine via lipid nanocapsules: preparation, statistical optimization, radiolabeling, in vivo biodistribution and pharmacokinetic study. Drug Deliv Transl Res 14(9):2539–2557. 10.1007/s13346-024-01528-7 38376620 10.1007/s13346-024-01528-7 PMC11525427

[CR47] Ibrahim TM, Abdulla NA, Mohamed MA (2024b) Investigating the efficacy of mirtazapine-embedded invasomal gel nanocarriers via I-optimal design for management of atopic dermatitis. J Drug Deliv Sci Technol 92:105395. 10.1016/j.jddst.2024.105395

[CR48] Jaudoin C, Agnely F, Nguyen Y, Ferrary E, Bochot A (2021) Nanocarriers for drug delivery to the inner ear: physicochemical key parameters, biodistribution, safety and efficacy. Int J Pharm 592:120038. 10.1016/j.ijpharm.2020.120038 33159985 10.1016/j.ijpharm.2020.120038

[CR49] Khoo X, Simons EJ, Chiang HH, Hickey JM, Sabharwal V, Pelton SI, Kohane DS (2013) Formulations for trans-tympanic antibiotic delivery. Biomaterials 34(4):1281–1288. 10.1016/j.biomaterials.2012.10.025 23146430 10.1016/j.biomaterials.2012.10.025 PMC3511665

[CR50] Lens M (2025) Niosomes as vesicular nanocarriers in cosmetics: characterisation, development and efficacy. Pharmaceutics. 10.3390/pharmaceutics17030287 40142950 10.3390/pharmaceutics17030287 PMC11946087

[CR51] Magdy M, Elmowafy E, El-Assal MIA, Ishak RAH (2022) Engineered triamcinolone acetonide loaded glycerosomes as a novel ear delivery system for the treatment of otitis media. Int J Pharm 628:122276. 10.1016/j.ijpharm.2022.122276 36270555 10.1016/j.ijpharm.2022.122276

[CR52] Mosallam S, Ragaie MH, Moftah NH, Elshafeey AH, Abdelbary AA (2021a) Use of novasomes as a vesicular carrier for improving the topical delivery of terconazole: in vitro characterization, in vivo assessment and exploratory clinical experimentation. Int J Nanomedicine 16:119–132. 10.2147/IJN.S287383 33447031 10.2147/IJN.S287383 PMC7802774

[CR53] Mosallam S, Sheta NM, Elshafeey AH, Abdelbary AA (2021b) Fabrication of highly deformable bilosomes for enhancing the topical delivery of terconazole: in vitro characterization, microbiological evaluation, and in vivo skin deposition study. AAPS PharmSciTech 22(2):74. 10.1208/s12249-021-01924-z 33586022 10.1208/s12249-021-01924-z

[CR54] Nemr AA, El-Mahrouk GM, Badie HA (2021) Development and evaluation of proniosomes to enhance the transdermal delivery of cilostazole and to ensure the safety of its application. Drug Dev Ind Pharm 47(3):403–415. 10.1080/03639045.2021.1890111 33625936 10.1080/03639045.2021.1890111

[CR55] Nemr AA, El-Mahrouk GM, Badie HA (2022) Hyaluronic acid-enriched bilosomes: an approach to enhance ocular delivery of agomelatine via D-optimal design: formulation, in vitro characterization, and in vivo pharmacodynamic evaluation in rabbits. Drug Deliv 29(1):2343–2356. 10.1080/10717544.2022.2100513 35869684 10.1080/10717544.2022.2100513 PMC9477486

[CR56] Nemr AA, El-Mahrouk GM, Badie HA (2023) Enhancement of ocular anti-glaucomic activity of agomelatine through fabrication of hyaluronic acid modified-elastosomes: formulation, statistical optimisation, in vitro characterisation, histopathological study, and in vivo assessment. J Microencapsul 40(6):423–441. 10.1080/02652048.2023.2215326 37192318 10.1080/02652048.2023.2215326

[CR57] Nemr AA, Ahmed S, Adel IM (2024) Limonene-enriched ultra-structural cubosomes to augment ocular delivery of a poorly water soluble anti-fungal drug: fabrication, characterization, statistical optimization, in vivo corneal uptake and histopathological evaluation in rabbits. J Drug Deliv Sci Technol 98:105886. 10.1016/j.jddst.2024.105886

[CR58] Sakr MG, El-Zahaby SA, Al-Mahallawi AM, Ghorab DM (2023) Fabrication of betaxolol hydrochloride-loaded highly permeable ocular bilosomes (HPOBs) to combat glaucoma: in vitro, ex vivo & in vivo characterizations. J Drug Deliv Sci Technol 82:104363. 10.1016/j.jddst.2023.104363

[CR59] Sayed S, Abdelmoteleb M, Amin MM, Khowessah OM (2020) Effect of formulation variables and gamma sterilization on transcorneal permeation and stability of proniosomal gels as ocular platforms for antiglaucomal drug. AAPS PharmSciTech 21(3):87. 10.1208/s12249-020-1626-2 32016607 10.1208/s12249-020-1626-2

[CR60] Sayed S, Abdel-Moteleb M, Amin MM, Khowessah OM (2021) Cubogel as potential platform for glaucoma management. Drug Deliv 28(1):293–305. 10.1080/10717544.2021.1872740 33509004 10.1080/10717544.2021.1872740 PMC7850357

[CR61] Singh A, Yadagiri G, Parvez S, Singh OP, Verma A, Sundar S, Mudavath SL (2020) Formulation, characterization and in vitro anti-leishmanial evaluation of amphotericin B loaded solid lipid nanoparticles coated with vitamin B12-stearic acid conjugate. Mater Sci Eng C Mater Biol Appl 117:111279. 10.1016/j.msec.2020.111279 32919641 10.1016/j.msec.2020.111279

[CR62] Su L, Zhao D, Huang Q, Zhao X, Chen Q, Rao H, Hao J (2024) Preparation of pectin-coated and chitosan-coated phenylethanoside liposomes: studies on characterization, stability, digestion and release behavior. Int J Biol Macromol 261(Pt 2):129442. 10.1016/j.ijbiomac.2024.129442 38232873 10.1016/j.ijbiomac.2024.129442

[CR63] Subroto E, Andoyo R, Indiarto R, Wulandari E, Wadhiah EFN (2022) Preparation of solid lipid nanoparticle-ferrous sulfate by double emulsion method based on fat rich in monolaurin and stearic acid. Nanomaterials. 10.3390/nano12173054 36080090 10.3390/nano12173054 PMC9457851

[CR64] Tawfik MA, Ahmed S, El-Dahmy RM, Aziz DE (2025) Oleic acid Enriched Leciplexes as novel mucoadhesive cationic nanocarriers of agomelatine for glaucoma treatment. AAPS PharmSciTech. 10.1208/s12249-025-03250-0 10.1208/s12249-025-03250-0 41136820

[CR65] Teama MT, Abdelmalak NS, Naguib MJ, Ahmed S (2025) Polymeric Micelles for Pulmonary Drug Delivery: A Comprehensive Review. BioNanoScience 15(3):504. 10.1007/s12668-025-02105-z

[CR66] Yang R, Wei T, Goldberg H, Wang W, Cullion K, Kohane DS (2017) Getting drugs across biological barriers. Adv Mater. 10.1002/adma.201606596 28752600 10.1002/adma.201606596 PMC5683089

[CR67] Younes NF, Habib BA (2022) Augmented local skin accumulation efficiency of sertaconazole nitrate via glycerosomal hydrogel: formulation, statistical optimization, ex vivo performance and in vivo penetration. J Drug Deliv Sci Technol 72:103364. 10.1016/j.jddst.2022.103364

[CR68] Younes NF, Sayed S, Hassan M, Ahmed S (2024) Engineered lecithin-based proniosomes for enhanced trans-tympanic permeation: in vitro, microbiological, ex vivo and in vivo evaluation. J Drug Deliv Sci Technol 96:105728. 10.1016/j.jddst.2024.105728

[CR69] Younes NF, Latif R, Badawi A, Hegazy K (2025) Optimized buccoadhesive repaglinide-loaded cubogel: in-vitro characterization and in-vivo hypoglycemic activity in a streptozotocin-induced diabetic rat model. International Journal of Pharmaceutics: X 10:100357. 10.1016/j.ijpx.2025.100357 40727682 10.1016/j.ijpx.2025.100357 PMC12302242

[CR70] Yousry C, Zikry PM, Basalious EB, El-Gazayerly ON (2020) Self-nanoemulsifying system optimization for higher terconazole solubilization and non-irritant ocular administration. Adv Pharm Bull 10(3):389–398. 10.34172/apb.2020.047 32665897 10.34172/apb.2020.047 PMC7335989

[CR71] Yousry C, Farrag NS, Amin AM (2023) Radiolabeling of statistically optimized nanosized atorvastatin suspension for liver targeting and extensive imaging of hepatocellular carcinoma. J Drug Deliv Sci Technol 80:104171. 10.1016/j.jddst.2023.104171

[CR72] Zhang Z, Li X, Zhang W, Kohane DS (2021) Drug delivery across barriers to the middle and inner ear. Adv Funct Mater. 10.1002/adfm.202008701 34795553 10.1002/adfm.202008701 PMC8594847

[CR73] Zhang T, Wu P, Owens G, Chen Z (2022) Adsorption and fenton-like oxidation of ofloxacin in wastewater using hybrid MOF bimetallic Fe/Ni nanoparticles. Chemosphere 307(Pt 2):135936. 10.1016/j.chemosphere.2022.135936 35934098 10.1016/j.chemosphere.2022.135936

[CR74] Zhu, J., Liu, B., Li, L., Zeng, Z., Zhao, W., Wang, G., & Guan, X. (2016). Simple and green fabrication of super-hydrophobic surface by one-step immersion for continuous oil/water separation. *The journal of physical chemistry. A, 120*. 10.1021/acs.jpca.6b06146 10.1021/acs.jpca.6b06146 27328269

[CR75] Zou J, Pyykko I, Hyttinen J (2016) Inner ear barriers to nanomedicine-augmented drug delivery and imaging. J Otol 11(4):165–177. 10.1016/j.joto.2016.11.002 29937826 10.1016/j.joto.2016.11.002 PMC6002620

